# Clinical practice of personalized prophylaxis in hemophilia: Illustrations of experiences and benefits from two continents

**DOI:** 10.1002/ccr3.2021

**Published:** 2019-02-20

**Authors:** Shannon Jackson, Cristina Santoro, Deborah Gue, Antonietta Ferretti, Domenico Gaglioti, Sandra Squire, Maria Gabriella Mazzucconi, Cedric Hermans

**Affiliations:** ^1^ Inherited Bleeding Disorders Program of BC – Adult Division St. Paul's Hospital Vancouver British Columbia Canada; ^2^ Division of Hematology, Department of Medicine University of British Columbia Vancouver British Columbia Canada; ^3^ Division of Hematology, Department of Cellular Biotechnologies and Hematology Sapienza University of Rome, Policlinico Umberto I Rome Italy; ^4^ Clinical Unit of Oral Lesion Surgery Sapienza University of Rome, George Eastman Institute, Policlinico Umberto I Rome Italy; ^5^ Hemostasis and Thrombosis Unit, Division of Hematology Cliniques Universitaires Saint Luc, Université Catholique de Louvain Brussels Belgium

**Keywords:** factor VIII, hemophilia, personalization, prophylaxis

## Abstract

These cases of people with hemophilia (PWH) illustrate the importance of understanding the patient's expectations and desires and adapting treatment to meet these needs, in addition to traditional clinical targets. Population PK modeling and FVIII products with improved PK profiles provide the opportunity to individualize care and improve long‐term outcomes.

## 1 INTRODUCTION

Hemophilia care is changing. The importance of individualizing care in improving long‐term outcomes and quality of life, and reducing direct and indirect costs of treatment, is increasingly recognized.^1^ At the same time, patient expectations have been transformed, both in the management of the disease and the desire for treatment to be compatible with their lifestyle choices. Prophylaxis is the standard of care for people with hemophilia (PWH),^2^ but there is now greater understanding that rigid prophylactic regimens do not take into account an individual's bleeding propensity, existing burden of joint damage, or pharmacokinetic (PK) response to the FVIII replacement used, and beyond the clinical aspects, their personal expectations and lifestyle requirements.

To overcome the large inter‐individual variability in PK responses to any given FVIII concentrate, and to provide individualized care, individual PK profiles should be generated for each PWH. However, the multiple measurements required are burdensome for patients, especially those who live a long distance from the Hemophilia Treatment Center (HTC) or who have significant mobility issues. Population PK modeling programs can predict an individual's PK profile, including trough factor levels, with fewer measurements than more traditional intensive sampling.^3^, ^4^ Combined with the availability of newer recombinant FVIII concentrates with improved PK profiles, these two advances provide the opportunity to further refine prophylactic options and improve patient outcomes.

At this time of rapid change in hemophilia care, it is more important than ever to share expertise and experiences across the globe. The following case reports are from Canada and Italy, two countries with extensive experience in providing prophylaxis for PWH. The cases illustrate the importance of understanding the patient's expectations and desires, and adapting treatment to meet these needs in addition to traditional clinical outcome targets.

## 2 CASE 1: AN OLDER CANADIAN MAN WITH AN ACTIVE LIFESTYLE

The patient is a 63‐year‐old retired teacher, with severe hemophilia A diagnosed at 8 months old. He has multi‐joint arthropathy, but no joint procedures, and a Hemophilia Joint Health Score (HJHS) of 44; in particular, the patient's right knee has been fused from multiple joint bleeds. He recovered from colon cancer in 1989, was diagnosed with vertebral osteomyelitis in 2004, and underwent eradication of hepatitis C in 2000, see Table 1 for his history. The patient is a keen tennis player and wishes to retain, or increase, his activity levels. This is partly to optimize his cardiovascular risk profile in the setting of a strong family history of coronary heart disease.

**Table 1:** Treatment history of an older Canadian man with an active lifestyle

Clinic visit	Treatment regimen	Patient observations	Codesigned approach
2008	On‐demand with rFVIII‐FS^a^	High level of bleeds, most of which required a follow‐up infusion	Standard prophylaxis
2011	Prophylaxis 2000 IU 3‐4 times weekly, rFVIII‐FS	Muscle and joints bleeds reduced but frequent joint swelling after exercise which requires an extra infusion to avoid a full‐blown bleed and use of NSAID	Individualized regimen of 1000 IU/d to cover exercise activities
2016	Transition to BAY 81‐8973^b^ 1000 IU/d	Transition was very smooth, with no issues	Maintain regimen
2017	BAY 81‐8973 1000 IU/d	Rarely thinks about bleeds, none reported. Daily prophylaxis has enabled him to feel as if he's gone “from a severe hemophiliac to a mild hemophiliac*”* His body has a more consistent chance to heal from little bumps and bruises. A solid foundation of daily prophylaxis has facilitated the use of NSAIDS on active days to manage pain and inflammation	Continue regimen ensuring appropriate use of NSAIDs

a rFVIII‐FS, sucrose‐formulated recombinant FVIII (Kogenate®, Bayer, Berkeley, CA, USA).

b BAY 81‐8973 (Kovaltry®, Bayer, Berkeley, CA, USA).

The patient participated in the Prophylaxis Clinic at the British Columbia Adult Bleeding Disorders Program at St. Paul's Hospital in Vancouver, in which an interdisciplinary team (IDT) engages patients in codesigning their own personalized regimen. Patient engagement requires the collection of outcomes from patients and sharing that information in the patient's preferred way (such as tables, graphs, verbal description) and allowing time for the patient to reflect. The British Columbia Prophylaxis Clinic provides a non‐clinical environment (no white coats, no equipment, etc) in which to conduct these patient reviews.

Through actively supporting patient autonomy in all aspects of decisions related to hemophilia management, the British Columbia Prophylaxis Clinic approach de‐emphasizes “adherence” as the primary goal. Instead of the traditional clinician focus on endpoints such as the annualized bleed rate (ABR), the Clinic focuses on a prophylaxis plan that is codesigned by the patient and aligned with his priorities.^5^ Adoption of this comprehensive team approach has been shown to reduce the ABR and improve patients' health‐related quality of life, independent of adherence to the prescribed prophylactic regimen.^6^

This patient was determined to be as physically active as possible to meet his goals, despite continuous pain. For example, despite being in pain for the first kilometer, he undertakes a daily 3 km walk to maintain his fitness, weight, and cardiovascular health. This highlights the importance of listening to the patient and not making assumptions about his needs or abilities. As a result of his transitioning to once‐daily prophylaxis, the patient is able to maintain a high level of physical activity and follows his clinician's guidance on the appropriate use of NSAIDs to reduce pain and inflammation on days of strenuous exercise.

The WFH guidelines for the management of hemophilia state that physical activity should be encouraged to promote physical fitness, with attention paid to muscle strengthening, coordination, general fitness, physical functioning, healthy body weight, and self‐esteem.^2^ Bone density per se may be decreased in PWH, and exercise is known to have a positive effect on bone mineral density in older people.^7^ In addition, exercise is an important part of weight maintenance which is of particular importance in this patient's target joint, as each pound of weight lost results in a four‐fold reduction in the load exerted on the knee per step during daily activities.^8^

The patient reports that he is very happy with his treatment regimen now, stating that daily prophylaxis makes him feel that he has gone “from a severe hemophiliac to a mild hemophiliac.”

## 3 CASE 2: A 30‐YEAR‐OLD CANADIAN MAN WITH STRONG TREATMENT REGIMEN PREFERENCES

A 30‐year‐old lawyer with severe hemophilia A (missense mutation) also participated in the British Columbia Prophylaxis Clinic. He had been on prophylaxis since undergoing immune tolerance induction (ITI) following an inhibitor at 6 months of age, Table 2. He has limited arthropathy, only the left elbow is affected, with a HJHS of three and no chronic pain. He has very defined preferences for his treatment regimen, being adamant that he did not want to infuse more than twice a week and not in the mornings. He reported that he wanted to increase his physical activity and spontaneity without adjusting his infusion schedule.

**Table 2:** Treatment history of a 30‐y‐old Canadian man with strong treatment regimen preferences

Clinic visit	Treatment regimen	Patient observations	Codesigned approach
2016	Prophylaxis with rFVIII‐FS^a^ 2000 IU twice weekly, on Monday and Fridays in the afternoon/evening	Desire to be more active and more spontaneous but to maintain his infusion schedule Infusion days = Activity days One ankle bleed in previous 12 mos	Previous population PK showed time to 1% = 88 h Switch infusion days to Tuesday and Friday to achieve a shorter interval between infusions (96 vs 120 h)
2017	Transition from rFVIII‐FS to BAY 81‐8973^b^ still at twice weekly dosing (Tue/Fri)	No further joint bleeds Increased physical activity	Updated population PK showing time to ~2% was 72 h and time to ~1% was 96 h

a rFVIII‐FS, sucrose‐formulated recombinant FVIII (Kogenate®, Bayer, Berkeley, CA, USA)

b BAY 81‐8973 (Kovaltry®, Bayer, Berkeley, CA, USA)

The patient associated “infusion days” with “activity days” but reported a desire to be more spontaneous about increasing his activity without increasing his infusion frequency. It is now understood that different lifestyles require different treatment thresholds. While a threshold of 1% is sufficient for patients with a sedentary lifestyle, the threshold for more active people with hemophilia is estimated at 3% for mild intensity, 5% for moderate intensity, and 10%+ for high intensity activities.^4^

In this patient, his post‐infusion time to 1% estimate (calculated using WAPPS, a web‐accessible, population modeling database that derives individual pharmacokinetic estimates from sparse samples; https://www.wapps-hemo.org/) increased by 8 hours after the change of FVIII concentrate and review of his infusion schedule. This allowed greater flexibility of times in which he could exercise, while adhering to his preference to not increase the frequency of infusions.

## 4 CASE 3: PROVIDING A “NORMAL” LIFE FOR A 7‐YEAR‐OLD ITALIAN BOY WITH SEVERE HEMOPHILIA A

The patient was diagnosed with severe hemophilia A at 11 months old and his treatment history is shown in Table 3. There was no prior family history of hemophilia. The parents were understandably concerned that their son should live as normal and active life as possible. Traveling to the clinic for infusions was costly and time‐consuming and limited the ability to adapt the dosing regimen to their son's active lifestyle.

**Table 3:** Treatment history of a 7‐y‐old Italian boy with severe hemophilia A

Clinic visit	Treatment regimen
Nov 2011	First infusion of rFVIII‐FS^a^ due to a minor head trauma
Nov 2011—Apr 2012	Nine further infusions of rFVIII‐FS for trauma and a hematoma
Apr 2012	Two doses of rFVIII‐FS due to a traumatic hemarthrosis of the right elbow The planned third dose not given because of difficult venous access Four doses of rFVIII‐FS after an early recurrence of the right elbow hemarthrosis a few days later
Apr 2012—Sep 2012	Once‐weekly prophylaxis initiated with 50 IU/kg of rFVIII‐FS
Sep 2012	Frequency increased to every 5 d due to elbow pain (dose remained at 50 IU/kg)
Nov 2012	Frequency increased to twice a week (50 IU/kg) due to ultrasound finding of mild/moderate synovitis in elbow
Nov 2013	No additional hemarthoses, but a few additional treatments for trauma Repeat ultrasound of the right elbow showed the continued presence of light/moderate synovitis Prophylaxis three times a week with the same dose of rFVIII‐FS, 50 IU/kg per dose, was initiated
Nov 2013—Sep 2015	Continued with rFVIII‐FS prophylaxis three times a week (50 IU/kg) No spontaneous bleeding One head trauma with a lacerated wound
Sep 2015	Following another right elbow hemarthrosis, prophylaxis of rFVIII‐FS was increased in frequency to alternate days at 40 UI/kg
Feb 2016	No bleeding problems Ultrasound of elbow showed only minimal synovitis
Jun 2017	Transitioned to BAY 81‐8973^b^; alternate days at 31 IU/kg No subsequent hemorrhagic problems PK data demonstrated higher trough levels with BAY 81‐8973 than with rFVIII‐FS (after 3 mos of treatment on alternate days) o BAY 81‐8973 31 IU/kg = trough level of 2.7% at 47 h after last infusion o rFVIII‐FS 33 IU/kg = trough level of 1.5% at 44 h after last infusion o rFVIII‐FS 31 IU/kg = trough level of 2.4% at 37 h after last infusion

a rFVIII‐FS, sucrose‐formulated recombinant FVIII (Kogenate®, Bayer, Berkeley, CA, USA)

b BAY 81‐8973 (Kovaltry®, Bayer, Berkeley, CA, USA)

When a child is diagnosed with severe hemophilia A with no family history, the parents have no prior experience with infusions. In this case, the parents wanted their son to enjoy a “normal” childhood but lived a considerable distance from the HTC. The provision of support services, such as the Bayer Patient Support Program in this instance, enabled them to learn how to provide infusions in the home setting, thereby facilitating more frequent infusions and the prevention of bleeds. Their son enjoys an active lifestyle, in particular regular swimming, which not only contributes to his quality of life but also plays an important role in his bone and general health.

A Swedish study highlighted the importance of a “normal” childhood for children with hemophilia, with many reporting that anxious parents and cautious teachers made them feel over‐protected, restricted in play activities and responsible for the burden of caring placed on their parents.^9^

## 5 CASE 4: SWITCHING TO PROPHYLAXIS LATER IN LIFE PROVIDES THE OPPORTUNITY FOR MORE COMPREHENSIVE CARE

A 60‐year‐old Italian man with severe hemophilia A had received on‐demand therapy all his life, since being diagnosed in childhood. He has a target joint (the right knee) and suffers from moderate iron‐deficiency anemia due to frequent gum bleeding as the result of an oral cyst and bad oral hygiene. He was diagnosed with HCV in 1993 but had not received treatment for this; see Table 4.

**Table 4:** Treatment history of a 60‐y‐old Italian man with severe hemophilia A

Clinic visit	Treatment regimen	Patient observations	Comorbidities/procedures
History	On‐demand (approximately 3 infusions/mo)	Resistance to prophylaxis due to venous access problems	Moderate iron‐deficiency anemia (Hb 9 g/dL); intolerance to oral iron therapy Frequent gum bleeding; no dental follow‐up HCV infection
Sep 2016	Short‐term prophylaxis with rFVIII‐FS^a^	Continued resistance to prophylaxis	Surgery for an inguinal hernia
Jun 2017	Twice‐weekly prophylaxis with BAY 81‐8973^b^	Venous access problems overcome with training from a patient support program	Intravenous iron therapy initiated Treatment for HCV initiated Dental appointment scheduled

a rFVIII‐FS, sucrose‐formulated recombinant FVIII (Kogenate®, Bayer, Berkeley, CA, USA)

b BAY 81‐8973 (Kovaltry®, Bayer, Berkeley, CA, USA)

The provision of dental treatment in patients with severe hemophilia A has often been neglected. In the 1960s, when this patient was a young man, the most common treatment pathway was extraction under general anesthesia followed by provision of dentures. As many general dental practices refuse patients with bleeding disorders, it is unsurprising that many PWH avoid the dentist until their treatment needs become severe and/or acute.^10^

Collaboration between the referral HTC and the dentist enabled a personalized protocol to be developed for this patient who underwent the successful removal of a cyst located in his lower jaw.

In this case, regular discussion with the patient provided a pathway to suggest a new treatment regimen that ultimately provided improved overall patient care. The two key factors in the patient's decision to switch from on‐demand to prophylaxis were (a) overcoming his venous access problems through the Patient Support Program and (b) the ability to provide protection with a low infusion frequency due to the longer time‐to‐trough with BAY 81‐8973. The patient now reports high satisfaction with his treatment, his anemia has been corrected (Hb 15 g/dL), he has had no further gum bleeding after his surgery, and there have been no intercurrent bleedings since the start of his prophylaxis.

## 6 DISCUSSION

These cases demonstrate the vital importance of engagement between hemophilia treaters and their patients to understand each individual's goals and philosophies, to define the shared objectives for the management of their hemophilia and optimize use of factor concentrates and other treatments, which is driven by a patient's understanding of their condition and the impact of these treatments on outcomes. Improvements to recombinant FVIII concentrates that result in improved PK profiles offer additional options to patients when adapting prophylaxis regimens.

In these cases, patients were transitioned from rFVIII‐FS (Kogenate®, Bayer, Berkeley, CA, USA) to BAY 81‐8973 (Kovaltry®, Bayer, Berkeley, CA, USA), which proved to be a smooth and uneventful process. BAY 81‐8973 is a full‐length, unmodified, recombinant human FVIII with the same primary amino acid sequence as rFVIII‐FS. Crossover PK studies have indicated a more favorable PK profile for BAY 81‐8973 than rFVIII‐FS or antihemophilic factor (recombinant) plasma/albumin‐free method (rAHF‐PFM; Advate®, Shire, Lexington, USA), supporting the potential for BAY 81‐8973 to provide a longer window of time above the FVIII trough level of 1%.^11^, ^12^ This potential is supported by simulations comparing typical patients on BAY 81‐8973 or rAHF‐PFM.^13^

Measuring a patient's PK profile can be burdensome, due to the blood draws from patients with many clinic visits required; however, population PK modeling tools, such as WAPPS, provide a means for HTCs to transition factor concentrates in a rigorous and objective manner. They also provide patient‐friendly information that help the patient to understand the implications of various prophylactic dosing strategies, to play an active part in making decisions about their treatment, and to adapt their treatment appropriately in the future.

As FVIII products with improved PK profiles become available, there is the potential to provide protection from bleeding with a reduced, or more flexible, infusion frequency. This may be a key factor in persuading on‐demand patients to switch to prophylaxis. For many patients, a change of FVIII concentrate represents a good opportunity for discussion about revision and adaption of treatment to better suit their individual needs.

Independence from the HTC for infusions may reduce direct costs, allows early treatment leading to better long‐term outcomes and improves patients' quality of life.^2^ Home support programs play a vital role in teaching successful home infusion techniques to patients and, where appropriate, their carers.

## 7 CONCLUSION

The management of severe hemophilia A should be built around each patient's lifestyle and needs to provide the best quality of life and the highest adherence to treatment. There should be timely re‐assessment of patients' needs and the available treatment options, with adaptation of their regimen as necessary. The case histories presented here illustrate some of the differing needs of patients suffering from the same condition in different health systems, and how those needs can be managed to provide optimal individualized patient care.

## CONFLICT OF INTEREST

None declared.

## AUTHOR CONTRIBUTION

Shannon Jackson: provide case information for cases 1 and 2, introduction, and discussion; Deborah Gue: provide case information for cases 1 and 2; Cristina Santoro: provide case information for case 3 and case 4; Sandra Squire: provide case information for cases 1 and 2, Antonietta Ferretti: provide case information for case 3 and case 4; Maria Gabriella Mazzucconi: provide case information for cases 3 and 4; Domenico Gaglioti: provide case information for case 4; and Cedric Hermans: provide the introduction and discussion. All authors contributed to the writing and reviewing the manuscript.
